# Services for Patients with Rare Genetic Diseases in Germany

**DOI:** 10.1007/s12687-026-00865-z

**Published:** 2026-01-31

**Authors:** Claudia Schmidtke, Peter Kühnen, Knut Mai, Christine Mundlos, Jakob Severin Cepus, Nicole Heider, Theresa Lipp

**Affiliations:** 1 Berlin Center for Rare Diseases, Charité-Universitaetsmedizin Berlin, corporate member of Freie Universität Berlin, Humboldt- Universität zu Berlin and Berlin Institute of Health, 13353 Berlin, Germany; 2 Alliance for Chronic Rare Diseases (ACHSE) e.V., Berlin, Germany; 3 German Centre for Child and Adolescent Health, DZKJ, Berlin, Germany; 4 Department for Pediatric Endocrinology and Diabetology, Charité-Universitätsmedizin Berlin, corporate member of Freie Universität Berlin, Humboldt- Universität zu Berlin and Berlin Institute of Health, 13353 Berlin, Germany; 5 Department of Endocrinology and Metabolism, European Reference Network on Rare Endocrine Diseases (ENDO-ERN), Charité-Universitätsmedizin Berlin, corporate member of Freie Universität Berlin, Humboldt- Universität zu Berlin and Berlin Institute of Health, 10117 Berlin, Germany; 6 Department of Human Nutrition, German Institute of Human Nutrition Potsdam-Rehbrücke, Nuthetal, Germany; 7 German Center for Diabetes Research (DZD), München-Neuherberg, Germany

**Keywords:** Rare Disease Centers Germany, National action plan, Patient pathways, Genomic medicine

## Abstract

It is estimated that there are currently around 4 million people with a diagnosed rare disease living in Germany, 300 million people worldwide. Rare Disease Centers (RDCs) were founded in Germany as part of the National Action Plan for People with Rare Diseases to improve patient care. Currently, more than 30 RDCs exist. With a focus on coordinating patient pathways and guiding people with a definite or a suspected diagnosis of a rare disease, RDCs organize interdisciplinary case conferences and form networks with dedicated local, national, and international experts for specific rare diseases. This article expounds on the current status and the function of RDCs in Germany. The Berlin Center for Rare Diseases will be discussed as an example. In addition to a description of the center’s workflow, its relationship to other organizations such as European Reference Networks and Patient Organizations as well as its role within the National Strategy for Genomic Medicine will be explained.

## Introduction

In the European Union, a disease is considered rare when it affects less than 5 in 10.000 people. 5.000 to 8.000 distinct rare diseases have been described so far. Currently, an estimated 4 million individuals living in Germany have been diagnosed with a rare disease (Ferreira 2019; Schlangen and Heuing 2023). Including close family members, such as parents and siblings, more than 10 million people are directly or indirectly affected by a rare disease in Germany.

Based on various activities of the European Organization for Rare Disease (EURORDIS), a European umbrella organization for people with rare diseases, the European Council took the initiative to address the challenges of rare diseases, which was preceded by the first French national plan for RD, starting as early as 2004 (Rodwell and Aymé 2015). In 2009, the European Council issued a recommendation to the European member states for a measure on rare diseases (European Council 2009). On the basis of this recommendation, the German Federal Ministry of Health (BMG), together with the Federal Ministry of Education and Research (BMBF) and the Alliance of Chronic Rare Diseases (ACHSE - Allianz Chronischer Seltener Erkrankungen e.V.), founded the National Action League for People with Rare Diseases (NAMSE) in 2010 (National Action League for People with Rare Diseases 2025). One of the main goals of NAMSE is to foster coordinated action by promoting collaboration within the German health care system. NAMSE brings together 28 partners who have jointly committed to improve health and quality of life of people with rare diseases in Germany. In 2013, after three years of coordination, the National Plan of Action for People with Rare Diseases was published. This plan, which is still in the process of implementation, consists of a number of measures in six fields of action that address the most pressing problems of those affected:

1. Care Centers, Networks.
2. Research.
3. Diagnostics.
4. Registries.
5. Information Management.
6. Patient Orientation (NAMSE Coordinating Office, 2013).

This article focuses on one cornerstone of the action plan: the establishment of specialized centers for rare diseases in Germany. As an example, the structures of the Berlin Center for Rare Diseases (BCSE) will be discussed in detail, exploring its evolving relationships to and role within European Reference Networks (ERNs), which were established when the German RD structures already existed, the National Strategy for Genomic Medicine (GenomeDE), and Patient Organizations (PO).

### RDCs in Germany

NAMSE recommended the implementation of three interconnected structures to enable and facilitate patient care for individuals with rare diseases, so-called type A, B, and C Centers.

Type A Centers, also referred to as Rare Disease Centers (RDCs), are reference centers affiliated with a university hospital. Currently, 36 such centers are established in Germany. Their main task is to guide both patients and family either with a diagnosed rare disease or an undiagnosed potential rare disease to specialized health care providers. In cases with undiagnosed diseases, RDCs use a standardized diagnostic approach to support patients and health care providers in the journey to diagnosis. Additional responsibilities include providing education on rare diseases, establishing diagnostic guidelines for rare diseases in cooperation with specialists, and supporting the implementation of specialized diagnostics (NAMSE Coordinating Office 2013).

While all RDCs in Germany share common goals and responsibilities, structures may vary from center to center. More information on specific RDCs can be found on SE-Atlas - Health Care Atlas for People with Rare Diseases, a web-based platform that offers information on care options (e.g. facilities, RDCs) in Germany (SE-Atlas 2025).

In accordance with the Plan of Action, RDCs have established networks. One of these is the Arbeitsgemeinschaft der Zentren für Seltene Erkrankungen (AG ZSE, RDCs Work Group). This network allows for information exchange on a national level. All German RDCs are represented in the AG ZSE, as well as the ACHSE (Hebestreit 2021; NAMSE Coordinating Office 2013).

Type B Centers provide care for specific diseases or groups of diseases. They are connected to a specific type A Center (RDC). Typically, type B Centers are part of clinics or departments within the university hospital. The number and specialization of these centers are determined by the locally available medical expertise (NAMSE Coordinating Office 2013).

Smaller centers, usually outpatient clinics, or practicing physicians contribute to the network as (disease specific) cooperating centers. These are the so-called type C Centers. They also supply specialized care either for a specific disease or multiple related diseases but are not associated with a university hospital (NAMSE Coordinating Office 2013).

### Certification process

A certification process was initiated for all established RDCs (type A Centers) in 2021 in order to ensure quality and expertise. RDCs are able to initiate the certification process with the independent certification agency ClarCert GmbH. Based on criteria of the NAMSE and the Federal Joint Committee (Gemeinsamer Bundesausschuss G-BA), the certification requirements include different aspects of quality control, e.g. case management, research, and networks. Currently over 20 RDCs have been certified.

### Berlin Center for Rare Diseases (BCSE)

The Berlin Center for Rare Diseases (BCSE) was founded in 2011 at the Charité-Universitaetsmedizin Berlin, Germany, and was successfully certified in 2024. The center combines pediatric and adult medical expertise in two teams under the heading of two chief physicians with the support of one coordinator. One team focuses on adult patients, aged 18 years and older and the other on pediatric patients, aged below 18 years. In addition to the chief physicians, both teams are comprised of guiding physicians, a qualified nurse, and medical students. The Institute of Medical Genetics and Human Genetics forms an essential part of the BCSE.

There are currently 19 type B Centers connected to the BCSE, 8 of which focus on pediatric patients, 8 on adult patients, and 3 provide care for patients of all ages. The central point of collaboration between the BCSE and its B centers is the joint care for patients with rare diseases. Additionally, they cooperate in providing visibility for rare diseases in the curriculum of the Charité-Universitaetsmedizin Berlin as well as mutually supporting academic endeavors and public events. It needs to be noted that not all disease groups are currently represented in B Centers connected to the BCSE. Care for patients with RD in the fields of ophthalmology, for example, is provided in (outpatient) clinics outside the BCSE. The BCSE also does not provide care in prenatal and preimplantation diagnostics (Table 1).

**Table 1:** Selection of associated specialist Centers/BCSE

Pediatrics	Adult Medicine	Pediatric and Adult Medicine
Amyloidosis Center Charité Berlin (ACCB)	Center for Mitochondrial Diseases	Christiane Herzog-Center for Cystic Fibrosis
Center for Cleft, Lip and Palate Surgery	Center for Rare Dermatological Diseases	Center for Rare Diseases in Audiology and Phoniatrics
Center for Primary Ciliary Dyskinesia	Center for Rare Connective Tissue and Edema Diseases	Institute of Medical and Human Genetics
Center for Rare Pediatric Endocrinological Diseases	Center for Rare Endocrinological Diseases	
Center for Rare Pediatric Gastroenterological, Nephrological and Metabolic Diseases	Center for Rare Hereditary Metabolic Diseases	
Center for Rare Pediatric Immunological and Infectious Diseases	Center for Rare Immunological Diseases, Immunodeficiencies and CFS	
Center for Rare Pediatric Oncological and Hematological Diseases	Center for Rare Rheumatological Diseases	
Center for Rare Pediatric Rheumatological and Autoimmune Diseases	Center for Rare Kidney Diseases (CeRKiD)	

Public awareness for the resources provided by RDCs is essential. Therefore, information on the BCSE can be found on its own website, through the SE-ATLAS- Health Care Atlas for People with Rare Diseases (SE-Atlas 2025), or through Orphanet, a web-based platform with information on rare diseases (e.g. classification of rare diseases, inventory of orphan drugs, directory of patient organizations) (Orphanet 2025). Furthermore, the BCSE contributes to activities celebrating the international annual rare disease days, collaborates with ACHSE, the national umbrella organization of patient advocacy groups, and engages in national and international conferences.

The BCSE plays an active role in the landscape of German RDCs. For example, it participates in frequent meetings with other RDCs through AG ZSE and is involved in other projects, such as the National Strategy for Genomic Medicine (genomeDE strategy) which will be expanded upon later in this article.

RDCs (e.g. BCSE) are financially supported to some extent by the health insurance system. Funding gaps nevertheless exist. The discussion on how to improve patients’ access to these vital services is ongoing, since sufficient financing of the NAMSE recommended structures is a cornerstone for optimal health care for patients with RD.

### Patient pathways into and within the BCSE

In order to access the resources of the BCSE, contact can be made via website, email, telephone, post, or fax. There are no restrictions placed on who can make the initial contact, e.g. the patients themselves, a family member, or a concerned friend. Patients can also be referred by physicians both outside and inside the Charité-Universitaetsmedizin Berlin, including B centers. This low threshold reflects the commitment of the BCSE to provide support for as many individuals afflicted with unknown or rare diseases as possible.

Once the initial contact is established, the team at the BCSE aids the person of contact and ultimately the patient in their journey to the health care provider(s) deemed to be the best suitable. Patient pathways at the BCSE are tailored to the patient’s position along the road to diagnosis at the initial contact.

If the patient has a confirmed diagnosis of a rare disease, he or she typically seeks specialists for their specific disease. In such cases, the contact to relevant specialists at the B Centers of the BCSE, at other specialized centers within Germany, or even abroad is established directly. If patients with a set diagnosis of a rare disease wish to establish contact with other patients or affected families, these patients are connected to ACHSE e.V., the patient umbrella organization or disease specific self-help groups.

If a rare disease is only suspected in a particular patient, a structural work-up algorithm has been established. The patient pathway is as follows:

Due to the high number of contacts from adult patients with unknown or undiagnosed disease, limitations have been placed on access to a thorough case evaluation. Due to high case load, only adult patients from Berlin and Brandenburg can submit their cases based on a physician-completed form detailing the medical suspicion of a rare disease. If patients live outside of Berlin or Brandenburg, they receive the contact information for their local RDC or the information for the SE-Atlas. For international patients we try to provide contact information for a RDC equivalent in their country or establish contact to Charité Healthcare Services GmbH, which facilitates appointments and case review for international patients at the Charité – Universitaetsmedizin Berlin. A complete medical history, including previous diagnostics and discharge letters, must be provided either by the patient him- or herself or by the physician. In contrast, acceptance of pediatric patients with unknown or undiagnosed diseases has no limitations placed upon it. No standardized questionnaire or physician evaluation must be completed for inclusion of pediatric patients. This is meant to lower the threshold for affected patients and their families.

Both Team Adults and Team Pediatrics thoroughly review the submitted case history and ensure that all necessary diagnostics for an all-encompassing systematic review of the case are present. If deemed necessary, the patient or physician is contacted to discuss any open questions or missing documents.

In Team Adults, once a thorough review of the patient history is completed, an appointment is made with the guiding physician. At this appointment, a detailed medical history is taken and a clinical examination performed. Based on the in-person evaluation and the previously provided data, the patients are categorized by urgency. Team Pediatrics does not require an in-person appointment as part of the case work-up, since inclusion of patients from all of Germany makes in-person appointments logistically more difficult.

Subsequently, in both teams, a full case review is performed, a summary compiled, and case relevant research completed. All relevant data is amassed in a presentation. These presentations are shared and the cases are discussed during interdisciplinary case conferences. The Team Adults meets biweekly with members of the case conferences including the following type B Center specialties: endocrinology, clinical genetics, rheumatology, dermatology and allergology, neurology, and psychosomatic medicine. Team Pediatrics holds weekly case conferences. Represented are physicians from pediatric endocrinology, pediatric immunology/pneumology, pediatric nephrology/gastroenterology/metabolic disease, pediatric neurology, and clinical genetics. Additional specialists are invited to discuss specific cases as needed. During the case conference, possible further diagnostic steps and differential diagnoses are deliberated and recommendations for the patient are formulated.

The patient is informed about these recommendations via a medical report that is transmitted by mail. Support for the implementation is provided by the BCSE. Since the aim of an RDC is not to perform diagnostics or to confirm a diagnosis, these steps are taken through the type B Center specialists. If further expertise on a disease specific basis is needed, type B Centers can collaborate with European centers of expertise for specific rare diseases or multiple related rare diseases through the European Reference Networks (ERNs). Complex cases can be discussed with international specialists to optimize diagnostics and treatments for patients with rare diseases. The Charité-Universitaetsmedizin Berlin is a reference center in 14 of 24 ERNs (European Reference Networks 2024). Further information on ERNs is given below.

Patients are requested to provide any diagnostic results stemming from the recommendations of the BCSE. If the results are unclear or a firm diagnosis cannot be made, a re-evaluation of the case with any newly provided information may be initiated by the BCSE. Once more, the case is discussed in an interdisciplinary case conference and differential diagnoses as well as recommendations for the patient are deliberated. The patient is informed of any new recommendations via another medical report. This process may be repeated several times.

The entire process is meant to aid patients in finding the right clinician(s) and expert(s) on their road to diagnosis and beyond. Additionally, patients receive a recommendation for contact with relevant patient networks. The aim is to make patient-relevant long-term care accessible to those affected by rare diseases.

### A new model project for genome sequencing in rare and oncological diseases

As of September 2024, a new patient pathway has become available to patients suffering from rare diseases through the “Model project for comprehensive diagnostics and therapy finding using genome sequencing for rare and oncological diseases” in accordance with § 64e SGB V, or Model Project Genome Sequencing (MPGS). This project enables clinicians to initiate whole genome sequencing (WGS) in patients with rare diseases with the aim of making a precise diagnosis and potentially initiating customized treatments (Model Project Genome Sequencing 2025). The framework is encased in the federal law and defined through a contract between the National Association of Statutory Health Insurance Funds (GKV-SV) and the Association of University Clinics Germany (VUD). These provide parameters to standardize genetic testing with set specifications for quality control (SGB V § 64e 2024, GKV-Spitzenverband 2024). Funds comprising 700 million Euros for this initiative have been made available by the statutory health insurance funds for a timeframe of five years, with the possibility for extending the project (Koordinationsstelle 2025).

RDCs play a key role in this initiative, since the presence of an RDC at the participating university hospital is a legal requirement for eligibility as a service provider (SGB V § 64e 2024). The contract between the GKV-SV and the VUD clearly delineates the role of RDCs in this project. One of the quality requirements states that WGS is initiated for a particular patient on the basis of a consensus decision of a case conference board (ZSE-Board). These ZSE-Boards include physicians from clinical genetics, other case relevant specialties, and the local RDC. The decision is based upon the medical history and clinical data pertaining to the patient. Relevant considerations in the decision-making process include whether WGS is deemed a medical necessity based on current scientific knowledge and whether added clinical value can result from the diagnostics. If it is expected that a medical benefit for the patient ensues from a WGS of one or both biological parents, the ZSE-Board can also recommend this analysis (GKV Spitzenverband 2024).

Once the diagnostics are completed, the ZSE-Board reconvenes to discuss the results of the case. Any necessary follow-up diagnostics are also determined. These can include segregation analysis, repeat phenotyping, functional testing, and establishing contact with research groups focusing on candidate genes (GKV Spitzenverband 2024).

At the Charité-Universitaetsmedizin Berlin, clinicians tending to patients with suspected monogenic rare diseases can initiate the inclusion of these patients in the model project, described below, through the BCSE or the Institute of Medical Genetics and Human Genetics. Both teams, along with the case clinicians, confer in the case conference regarding the patients and reach the consensus decision to initiate whole genome diagnostics. After genome sequencing and analysis, the second case conference, under the participation of the case clinicians, the BCSE, the geneticists, and any other case relevant specialists, occurs. Here the possible genetic variants are discussed and interpreted and a plan is established in case any further diagnostics is deemed necessary by the team.

### Patient Organizations

Shortcomings in the healthcare and social care systems often prompt patients to take action on their own behalf. This is particularly true for those affected by rare diseases and their relatives. The rarity of their diseases sends them on a quest: first for the correct diagnosis, including an odyssey from doctor to doctor, then for disease-specific information and suitable experts who can contribute the necessary knowledge and experience for the treatment and long-term care. Patients and their relatives repeatedly experience rejection and incomprehension along the way because knowledge of these diseases is not widespread in the medical profession and the presence of a rare disease is rarely taken into consideration (EurordisCare 2009).

Once the diagnosis is established, PLWRD (people living with rare diseases) are still required to advocate for the issues associated with their disease and to deal with the lack of specialists and specialized outpatient clinics, effective therapies, lay accessible information, and sound knowledge. At this point, they often join or form a disease-specific patient organization - to gather relevant information and share knowledge, and to gain understanding, concrete help and empowerment in coping and living with the disease through exchange with others affected. Also politically, rare disease patient organizations are committed to achieving equal access to medical, nursing, and social care for those affected (Hoffmann et al. 2020).

This encouraged some representatives of Rare Disease patient organizations to found an umbrella organization, the Alliance of Chronic Rare Diseases - ACHSE, in 2004 from within a working group of the Federal Working Group of Self-Help (BAG-SH). Today, over 140 patient organizations representing one or more rare diseases are united within ACHSE. ACHSE acts as a multiplicator and mediator for the network and raises awareness in the general public as well as in specific target groups and stakeholders to the overarching needs of PLWRD. It voices affected people’s needs and represents their interests in politics and the healthcare system – including on the European level (Biehl et al. 2021).

Specifically, ACHSE contributes to improvements with the following measures:

- counseling/a help line for PLWRD seeking advice, but also for doctors, therapists, scientists – supporting other interested stakeholder in dealing with a rare disease.
- preparing and disseminating quality-assured information.
- building a network within the health and social care system.
- initiating and cooperating in research projects.
- contributing to and further developing self-help activities through joint cooperations within the self-help network.

Until the establishment of the centers described above in this publication, ACHSE was the only nationwide contact point for PLWRD and for those with an unclear diagnosis. In 2008, ACHSE also initiated the first national medical contact point for enquirers for health care professionals and scientific staff.

The collaboration between the ACHSE and the RDCs has proven to be crucial for improving the care of people with rare diseases. ACHSE serves as a central point of contact for affected individuals, offering comprehensive counseling to reduce uncertainties following a diagnosis.

ACHSE representatives actively participate in pilot meetings and training sessions of RDCs. This fosters an exchange between medical experts and patient representatives, ensuring that the needs of patients are better integrated into healthcare structures.

Direct communication between ACHSE and the staff of the centers enables a productive exchange regarding the concerns of those seeking advice. This collaboration helps e.g. to identify medical experts for specific rare diseases, thereby enhancing diagnostic and treatment processes.

ACHSE and the RDCs work together to establish quality standards for the care of PLWRD. By establishing quality criteria and implementing certification procedures, they ensure that high standards of care are consistently maintained.

This partnership significantly enhances the quality of care through structured networks that enable faster diagnoses and improved treatment options. By pooling expertise and incorporating the patient perspective, this collaboration plays a key role in improving the quality of life for people with rare diseases.

Furthermore, ACHSE was and still is involved in the ongoing process of further development of RDCs, e.g. through part taking in projects such as TRANSLATE-NAMSE (Rillig et al. 2022) and ZSE-DUO (Hebestreit et al. 2022) on the organization of care at type A centers and is also a project partner in the ongoing Innofonds project B(e)NAMSE (B(e) NAMSE 2025), which focuses on the care of children, adolescents and young adults on the type B center level.

In cooperation with the RDCs, ACHSE has been organizing a national conference (NAKSE) every two years since 2019, which is attended by up to 500 stakeholders from medicine, healthcare, politics, and representatives of patient organizations. Patient advocates from ACHSE member organizations also engage actively in European Reference Networks (ERNs) as part of their European Patient Advocacy Groups, benefiting from and contributing to a vivid Rare Disease Community among patients and HCPs, across disciplines and national borders.

At the European level, ACHSE members are active in disease-specific ERNs through participation in the European Patient Advocacy Groups (ePAG). The experience gained from these work processes are pooled in an ACHSE working group and used to establish a structure for future national cooperation between B-level-RDCs and disease-specific patient organizations.

### European Reference Networks

Within Europe, networks have been founded to improve the care of people living with a rare disease.

The European Reference Networks (ERNs) were established in order to combine the expertise of European hospital centers aiming to improve the situation and health care for patients with rare diseases on several levels. In 2024, 1,619 specialized centers localized in 382 hospitals across 27 Member States (and including Norway) were part of 24 ERNs. The selection of ERNs is based on disease areas relevant for the particular case. This allows all specialists to discuss certain complex cases within a consortium and receive advice to establish an optimal diagnostic and treatment approach. Virtual expert discussions are regularly performed via the platform Clinical Patients Management System (CPMS). Additionally, ERN provides the development of clinical practice guidelines, training, and education activities and is engaged to establish registries of rare diseases on a European level. Collaboratively, the ERN members exchange knowledge, experience, and expertise to support colleagues within member states to make the best care available for patients with rare diseases. The input and participation of patient advocacy groups (PAG) is one key aspect to improving this network. (European Reference Networks 2024)

This ERN-collaboration has been developed somehow independently from the NAMSE rare disease infrastructure in Germany (NAMSE A- and B-centres), which might have led to difficulties to align the strategic aims of both initiatives. This is exemplified by the observation that not all NAMSE B-Centres in Germany are certified as ERN centres. It might additionally be related to the observation that national health care systems in European countries differ if and how they asign centres of excellence for specific rare diseases. Therefore, it remains an ongoing task and responsibility to find a solution to adjust the national action plan in Germany to the ERN requirements and conceptional objectives. It would lead to less misconception, confusion and reduce additional expenses, if redundant processes within the health care system would be eliminated.

## Discussion and conclusions

The National Action League for People with Rare Diseases with its action plan is the cornerstone of many health care structures for PLWRD in Germany, some of which are still in the process of implementation. One outcome of these structures is the formation of Rare Disease Centers. For people with a diagnosed rare disease, a well-structured network of Type B and Type C centers helps to find the correct expertise and care fast and without many burdens (as closest to where the patient lives). For people with unknown diseases, interdisciplinary collaborations (case conferences) help to guide the patient towards specific diagnostics in order to get a definite diagnosis quickly.

At the same time, patient organizations play an important role as supporting services for PLWRD.

National projects such as TRANSLATE-NAMSE demonstrate the benefits of a multidisciplinary and interconnected center model, including interdisciplinary case conferences as a tool to help undiagnosed patients. The demand for people with unknown, suspected rare diseases to have some sort of contact point, when the standard care was exhausted, was and continues to be a significant issue. Although inroads have been made, there remains room for improvement. The increasing number of RDCs in Germany as well as increasing numbers of enquiries within the RDCs highlight the importance of these coordinating and disease specific centers for people with suspected or diagnosed rare diseases.

Due to the fact that the model for RDCs under the NAMSE action plan is nationally implemented, patients all throughout the country can access care. Yet, when looking at the map of RDCs in Germany, it can be noted that RDCs are sparse in less populous regions of the country that have fewer medical universities (SE-Atlas 2025) Patients in these regions therefore have a higher threshold for receiving assistance through an RDC. Furthermore, due to the limited financial resources, the aforementioned structures are not able to provide comprehensive services to all patient that require specialized treatments, aggravated by the fact that the demand for care for PLWRD is increasing due to progress in basic research and its translation into diagnostics and therapies.

The growing demand for care for PLWRD is met by a number of ongoing activities, which includes the model project with its rooting in the national social lawbook, SGB V, described above.

The AG-ZSE allows for frequent information exchange between type A Centers on a national level. Yet, no unified patient intake and communication system has been established so far. To meet this need, at the level of a Bundesland (federal state), the Bavarian RDCs have established Bayrischer Arbeitskreis für Seltene Erkrankungen (BASE-Netz, Bavarian Work Group for Rare Diseases). BASE-Netz simplifies communication between separate RDCs, which streamlines casework and supports the collaboration between centers (BASE Netz 2025). Due to the specificity of expertise required, a similar construct would be helpful for all German RDCs. Initiatives regarding interconnectivity have been instigated.

While type B Centers already communicate regularly with international experts through the ERNs, this approach does not account for patients with undiagnosed diseases affecting multiple organ systems and hence requiring multiple areas of expertise. Therefore, further networks are required, such as undiagnosed disease programs connecting type A RDCs, for which the Undiagnosed Diseases Network International (Taruscio et al. 2020) may serve as a blueprint.

RDCs in Germany stand out in terms of accessibility, expertise, and networking. While a high level of expertise is available on a regional level and cooperation between RDCs exist, these can be further improved upon. Additionally, the already existing partnerships with ACHSE and specific Patient organizations can further be strengthened to better advocate for PLWRD. On an international level, similarities between RDC structures, where existing, can be used as a basis for collaboration.

## Data Availability

No datasets were generated or analysed during the current study.

## Abbreviations

ACHSE: Allianz Chronischer Seltener Erkrankungen e.V. (Alliance for Chronic Rare Diseases)
BCSE: Berliner Centrum für Seltene Erkrankungen (Berlin Center for Rare Diseases)
BMBF: Bundesministerium für Bildung und Forschung (Federal Ministry of Education and Research)
BMG: Bundesministerium für Gesundheit (Federal Ministry of Health)
CPMS: Clinical Patients Management System
ePAG: European Patient Advocacy Groups
ERN: European Reference Networks
EURORDIS: European Organization for Rare Diseases
GenomeDE: National Strategy for Genomic Medicine
GKV-SV: Gesetzliche Krankenversicherung – Spitzenverband (National Association of Statutory Health Insurance Funds)
MPGS: Model Project Genome Sequencing
NAMSE: National Action League for People with Rare Diseases
PAG: Patient Advocacy Groups
PLWRD: People Living with Rare Diseases
PO: Patient Organizations
RDC: Rare Disease Center
VUD: Verband Universitätsklinika Deutschland (Association of University Clinics Germany)
WGS: Whole genome sequencing
